# Sugar Metabolism in Hummingbirds and Nectar Bats

**DOI:** 10.3390/nu9070743

**Published:** 2017-07-12

**Authors:** Raul K. Suarez, Kenneth C. Welch

**Affiliations:** 1 Department of Zoology, University of British Columbia, #4200-6270 University Blvd., Vancouver, BC V6T 1Z4, Canada; 2 Department of Biological Sciences, University of Toronto, 1265 Military Trail, Scarborough, ON M1C 1A4, Canada; kwelch@utsc.utoronto.ca

**Keywords:** sugar, glucose transport, hexokinase, metabolism, muscle, energetics, evolution, foraging behavior

## Abstract

Hummingbirds and nectar bats coevolved with the plants they visit to feed on floral nectars rich in sugars. The extremely high metabolic costs imposed by small size and hovering flight in combination with reliance upon sugars as their main source of dietary calories resulted in convergent evolution of a suite of structural and functional traits. These allow high rates of aerobic energy metabolism in the flight muscles, fueled almost entirely by the oxidation of dietary sugars, during flight. High intestinal sucrase activities enable high rates of sucrose hydrolysis. Intestinal absorption of glucose and fructose occurs mainly through a paracellular pathway. In the fasted state, energy metabolism during flight relies on the oxidation of fat synthesized from previously-ingested sugar. During repeated bouts of hover-feeding, the enhanced digestive capacities, in combination with high capacities for sugar transport and oxidation in the flight muscles, allow the operation of the “sugar oxidation cascade”, the pathway by which dietary sugars are directly oxidized by flight muscles during exercise. It is suggested that the potentially harmful effects of nectar diets are prevented by locomotory exercise, just as in human hunter-gatherers who consume large quantities of honey.

## 1. Introduction

Hummingbirds and nectar bats became nectarivorous animals in a process that involved coevolution with the flowering plants offering them nectar [1]. As their diets, foraging and feeding modes evolved, so did the suite of morphological, physiological and biochemical traits that made them adapted for “aerial refueling” [2,3,4]. Hummingbirds rely mainly on the sugars in floral nectar to fuel their high metabolic rates [5]. Perhaps less widely known is that nectar bats also derive most of their dietary calories from sugars [4]. Some nectar bat species can hover while feeding [6], behaving as “hummingbirds of the night”. The features allowing hover-feeding in hummingbirds and nectar bats are remarkable examples of convergent evolution. This review serves as a primer on their sugar metabolism. As such, the intention is not a comprehensive review of the literature but, rather, a more focused introduction to aspects of their sugar metabolism, particularly in relation to exercise, presented in an evolutionary and ecological framework. Most of the discussion shall be based on data obtained from hummingbird species of between 3 to 5 g in body mass and from 10 g Pallas’ long-tongued nectar bats (*Glossophaga soricina*). The findings summarized here offer opportunities for comparison with *Homo sapiens*, a species that is unable to rely to the same extent on the direct oxidation of dietary sugar to fuel exercise and that suffers from the adverse effects of excessive sugar ingestion.

## 2. Diet and Digestion

**Figure 1:**
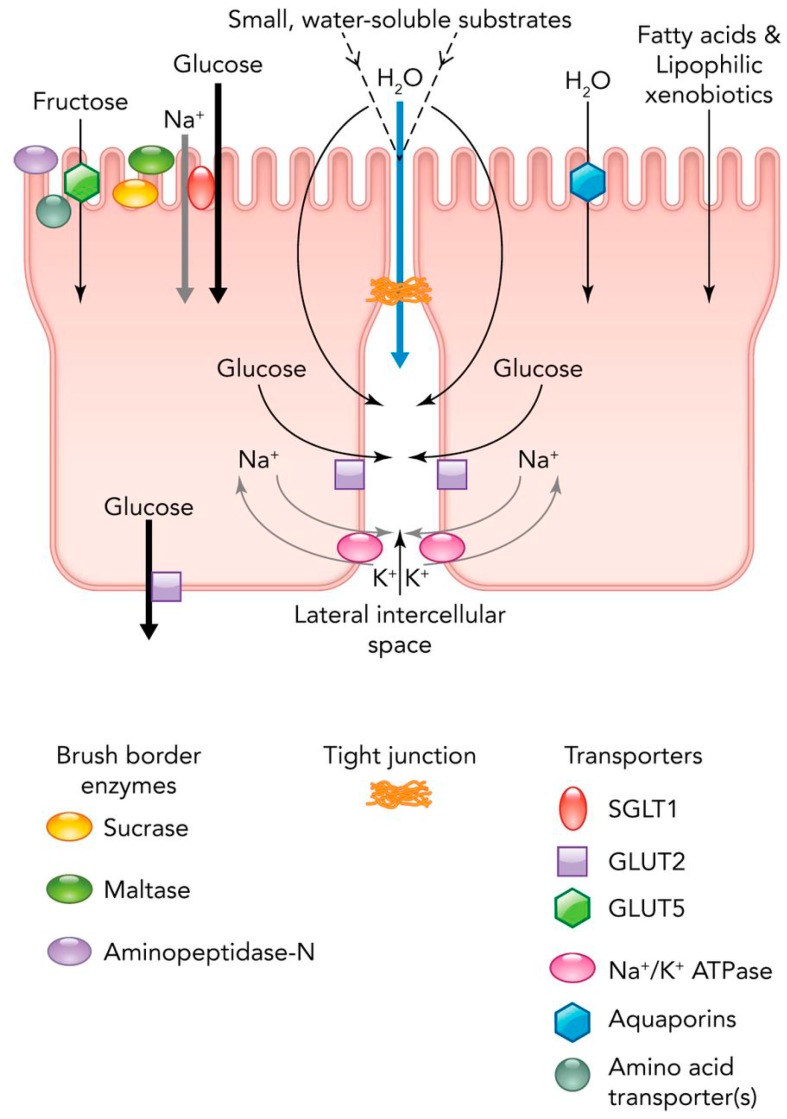
A model of the principal mechanisms by which nutrients are absorbed across the avian and chiropteran intestinal border. While both fructose and glucose are absorbed at high rates across the brush border via carrier-mediated pathways, as occurs in humans and other terrestrial mammals, substantial flux occurs via paracellular (diffusion or solvent drag) pathways in flying vertebrates [1]. Among small nectarivore species, like hummingbirds and nectar bats, brush border enzyme and glucose transporter (GLUT) and Na^+^-dependent glucose transporter (SGLT)-mediated transport activity per unit of intestinal area is high. However, paracellular absorption must also occur at especially high rates in the intestines of these nectarivores in order to satisfy overall energy budget (and thus absorptive) demands. Figure reprinted with permission from Price et al. [19].

The flowering plants visited by hummingbirds and nectar bats evolved as “prey that want to be eaten” [7,8] that benefit from the pollination services provided by these animals in exchange for the sugars they produce. In the course of their coevolution with flowering plants, three major groups of birds (hummingbirds, honeyeaters and sunbirds) [9] and two groups of phyllostomid bats (Lonchophyllinae and Glossophaginae) [10] adopted nectarivorous diets. While frugivorous birds generally ingest fruits rich in glucose and fructose, but not in sucrose [11], hummingbirds preferentially ingest sucrose-rich nectars that contain less glucose and fructose [8]. Nectar bats ingest sugar mixtures in fruits and nectars that are rich in these monosaccharides, but low in sucrose [11]. However, nectar bats are able to vary their degree of reliance on fruit pulp and floral nectar according to availability [12]. The dietary specialization of hummingbirds is made possible by expression of high levels of intestinal sucrase [13], a trait not found in many species of frugivorous birds. In addition, hummingbird intestines in vitro display the highest known rates of intestinal active transport of glucose [14,15]. However, the maximum capacity for active transport of glucose is far below the physiological rate at which sucrose is assimilated in vivo [5,14]. Instead, a paracellular transport mechanism accounts for most of the movement of sugar across the intestinal epithelium [16] (Figure 1). Nectar bats also have high levels of intestinal sucrase, allowing hydrolysis of sucrose contained in nectars and fruits [17], and make use of a predominantly paracellular pathway for intestinal sugar absorption [18]. Hummingbirds and nectar bats ingesting sugars display digestive efficiencies close to 100% [12,15].

**Figure 2:**
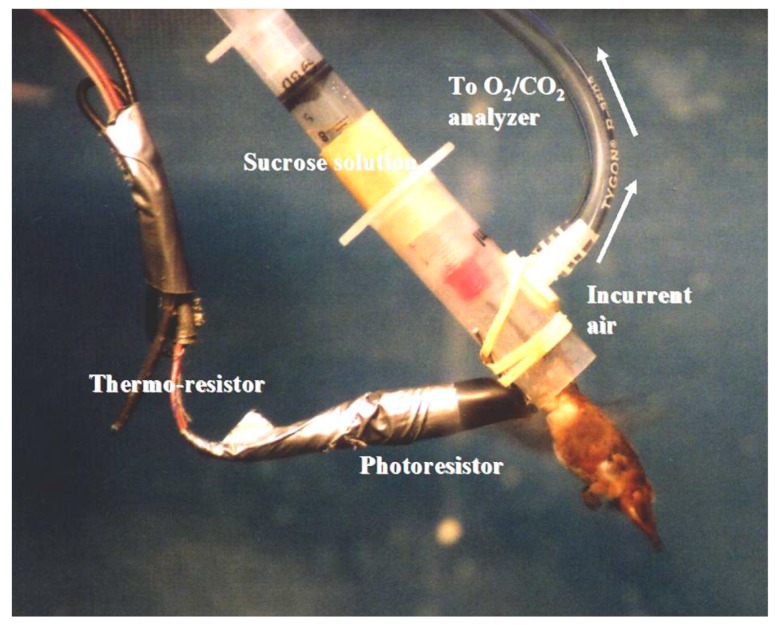
Hummingbird mask respirometry. The bird is freely hovering while feeding on a sucrose solution with its head in feeder modified to function as a mask for flow-through respirometry. Air is drawn into the mask at a known flow rate. The air, depleted of O_2_ and enriched with CO_2_, is analyzed downstream using O_2_ and CO_2_ analyzers. See [20,26] for detailed description of the method.

Feeding on floral nectar while hovering requires extremely high rates of energy expenditure. These are most commonly measured under laboratory conditions and in the field using mask-respirometry [6,20] (Figure 2). Small hummingbirds in routine hovering flight display wingbeat frequencies of 30–60 Hz [21,22] and, in the process, sustain the highest mass-specific rates of aerobic metabolism among vertebrates that are about tenfold higher than the maximum rates measured in human athletes [2]. Ten-gram nectar bats (*Glossophaga soricina*) beat their wings at lower frequencies (9 Hz) [23] and display hovering mass-specific metabolic rates [6,24] about half those of hummingbirds. Nevertheless, these approximate the mass-specific metabolic rates of shrews exposed to low ambient temperature [25] and are among the highest values recorded among mammals.

The need to fuel such high metabolic rates raises interesting and important questions concerning the fate of ingested sugars. At high exercise intensities, 90% or more of whole body O_2_ consumption rates are accounted for by mitochondrial respiration in exercising muscles [2,27]. Decades ago, recognition of the importance of fat as the main fuel stored before and depleted during avian migration led to the idea that bird flight muscles use mainly fatty acid oxidation as their source of ATP during exercise [28]. The nectarivorous diet of hummingbirds and nectar bats therefore raises the question of whether their energy metabolism during flight might be fueled primarily by ingested sugar or, alternatively, by fat previously synthesized from ingested sugar. A third possibility is that ingested sugar is used for the synthesis of glycogen, which is then broken down to fuel metabolism during flight.

## 3. Biochemical Capacities for Substrate Oxidation

In their invasion of a niche previously occupied by insects, hummingbirds and hovering nectar bats evolved large pectoral muscles relative to total body mass. These consist exclusively of fast-twitch, oxidative fibers [29,30,31] that possess high mitochondrial content [30,32]. The high O_2_ requirements during exercise are supported by high lung O_2_ transport capacities [33,34], large hearts [35,36] and high muscle capillary densities [37]. In rufous hummingbird (*Selasphorus rufus*) flight muscle fibers, mitochondria occupy 35% of cell volume and respiratory capacities are further enhanced by cristae surface densities (cristae surface area/mitochondrial volume) about twofold higher than those found in mammalian muscle mitochondria [30]. Enzymatic capacities for substrate oxidation are enhanced, as indicated by *V_max_* values (=*k*_cat_ × [*E*], where *k_cat_* is catalytic efficiency and [*E*] is enzyme concentration), measured in vitro (Table 1). Consistent with the high mitochondrial content of these muscles are their high *V_max_* values for the Krebs cycle enzyme, citrate synthase. High capacities for glucose phosphorylation and fatty acid oxidation are indicated by high *V_max_* values for hexokinase and carnitine palmitoyl transferase, respectively. It is important to point out that, although *V_max_* values establish upper limits to flux [38,39], they do not serve as measures of physiological rates through metabolic pathways in vivo. Which fuels are oxidized, at what rates and under what circumstances are empirical questions that must be addressed using other approaches [39,40].

## 4. Substrate Oxidation during Foraging Flights

Reaction to the suggestion that nectarivorous animals might directly fuel their metabolism during exercise using dietary sugar is often, “Of course—what else would one expect?” On the contrary, it is well known among exercise physiologists and biochemists that rates of glucose phosphorylation in most vertebrate skeletal muscles are insufficient to account for the metabolic rates required during high-intensity exercise [40,42]. Hexokinase *V_max_* values in vertebrate muscles are generally low [43] (Table 1). In most species during exercise, hexokinase operates at very low fractional velocities (*v*/*V_max_*) [40], limiting entry of glucose into the glycolytic pathway in muscles [44]. Fell [45] goes as far as to disqualify hexokinase as a glycolytic enzyme but, rather, considers the reaction it catalyzes to be primarily involved in the synthesis of glycogen. As exercise intensities increase, the reliance on fatty acid oxidation in mammalian muscles declines and carbohydrate oxidation becomes the greater contributor to the fueling of energy metabolism [46,47]. Since, under these conditions, glucose phosphorylation rates are insufficient to match the rates of carbohydrate oxidation observed, glycogenolysis provides most of the carbon oxidized during exercise as maximum aerobic metabolic rates (V${}_{O_{2}}$*_max_* values) are approached [42,47]. What might seem so obviously true to some would therefore appear highly unlikely to those familiar with metabolism during exercise in mice, rats and humans. The contrast between preconceived notions and these empirical results makes the subject of sugar metabolism in hummingbirds and nectar bats all the more interesting.

**Figure 3:**
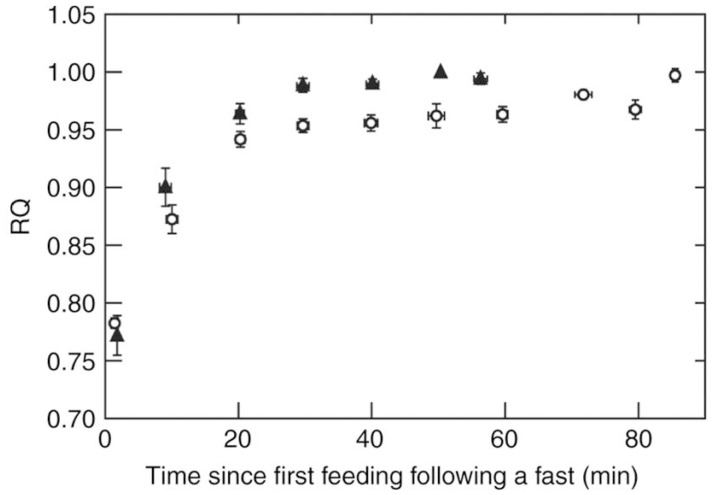
Respiratory quotients (RQ) during hover-feeding over time after fasting in rufous hummingbirds (*Selasphorus rufus*) (triangles) and nectar bats (*Glossophaga soricina*) (circles) (From [3]). Flight muscles oxidize mainly fat (RQ values close to 0.7) in fasted animals during hovering. RQs rise to about 1.0, indicating that flight muscles shift to carbohydrate oxidation as a result of repeated hover-feeding on sucrose solutions.

Respiratory Exchange Ratios (RER = Vc${}_{O_{2}}$/V${}_{O_{2}}$), measured using mask respirometry [20] (Figure 2), in these animals are considered to closely reflect cellular Respiratory Quotients (RQ = Vc${}_{O_{2}}$/V${}_{O_{2}}$). This is likely to be the case: a 4-g hummingbird with a blood volume of 0.4 mL, carrying 0.088 mL O_2_ [48], respires at a rate of about 2 mL O_2_ per minute [30]. At this metabolic rate, blood O_2_ stores would be completely depleted in 2.6 s if whole-body O_2_ uptake and mitochondrial respiration were not tightly linked. The rate of mitochondrial respiration in the flight muscles during hovering is so high and so closely coupled to whole-body gas exchange rate that even substrate-dependent differences in moles of ATP synthesized per mole of O atom consumed [49,50] can be detected using respirometry [51]. Measured Vc${}_{O_{2}}$/V${}_{O_{2}}$ values shall henceforth be referred to as RQs to facilitate biochemical interpretation. Fasted hummingbirds and nectar bats, perched or hanging upside down, display RQ values of about 0.7, indicating that fatty acid oxidation fuels their whole-body resting metabolic rates [52,53,54]. Under resting conditions, energetically expensive internal organs account for most of the whole-body metabolic rate while skeletal muscles account for only a small fraction. When they fly to forage for food, whole-body metabolic rates increase dramatically and the high V${}_{O_{2}}$ values, measured using mask respirometry, are mainly due to the flight muscles. Repeated hover-feeding bouts and ingestion of sugar solutions result in progressive increases in RQ values to about 1.0 [52,53,54] (Figure 3). This indicates that the flight muscles progressively rely more on carbohydrate oxidation as sugar is repeatedly ingested.

**Figure 4:**
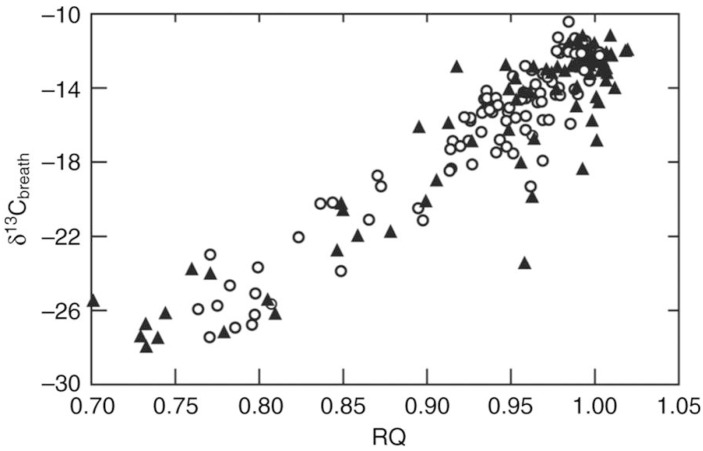
δ^13^C of expired CO_2_ as a function of RQ in hover-feeding rufous hummingbirds (*Selasphorus rufus*) (triangles) and nectar bats (*Glossophaga soricina*) (circles) (From [3]). More negative δ^13^C values characteristic of maintenance beet sugar are observed when animals are hovering in the fasted state with RQ values close to 0.7. As RQ values rise to 1.0, indicating transition from fat oxidation to carbohydrate oxidation, δ^13^C values also increase to approximate δ^13^C of cane sugar provided in feeders during mask respirometry experiments.

The nature of the carbohydrate oxidized during hover-feeding flights was revealed by combining the use of carbon stable isotopes with mask respirometry. Beet-derived sucrose, produced by C3 photosynthesis, is relatively more ^13^C-depleted than cane-derived sucrose, the product of C4 photosynthesis [55]. Measured as δ^13^C, where

(1) $$\delta^{13}C=\frac{\left[{}^{13}C\right]/\left[{}^{12}C\right]}{R_{std}}$$

and *R_std_* is a standard [56], a more negative δ^13^C value would be expected upon analysis of CO_2_ expired by animals maintained on beet-derived sucrose compared with the CO_2_ produced by animals maintained on cane-derived sucrose. In these experiments, animals were first maintained on diets containing beet-derived sucrose until they expired CO_2_ with δ^13^C values similar to that of beets. Animals were then fasted until RQ = 0.7, then given free access to feeders fitted with masks to allow sampling of expired CO_2_ as well as measurement of V${}_{O_{2}}$ and Vc${}_{O_{2}}$ during hovering flight. Figure 4 shows that as the hummingbirds and nectar bats engaged in the first feeding bouts, RQ values were close to 0.7, indicating that their flight muscles oxidized mainly fat. As they made repeated hovering visits to the feeder and fed on sucrose solutions, the RQ values rapidly approached 1.0, while the δ^13^C of their expired CO_2_ rose from the more negative values characteristic of beet sucrose to less negative values characteristic of cane sucrose. It can be inferred from these results that the increase in RQ, i.e., the switch from fat oxidation to carbohydrate oxidation, represents a transition from the oxidation of endogenous fat to dietary sucrose by the flight muscles. Fasted animals that oxidize fat (synthesized from beet sucrose) rapidly switch to oxidizing cane sucrose to fuel their energetically expensive hovering flight soon after they start feeding on cane sucrose. While humans can directly fuel about 30%, at most, of exercise metabolism with ingested glucose and fructose [57], in hummingbirds and nectar bats, the contributions of recently-ingested sucrose to energy metabolism during hovering are about 95% and 80%, respectively [3]. The carbon stable isotope results obtained using the protocol outlined here are in general agreement with those obtained independently by Voigt and colleagues using a different approach [58].

The *V_max_* values for hexokinase and carnitine palmitoly transferase in both hummingbird and nectar bat flight muscles (Table 1) indicate that catalytic capacities at these steps in both species are sufficient to account for the rates of glucose and fatty acid oxidation estimated during hovering flight (Table 2).

**Figure 5:**
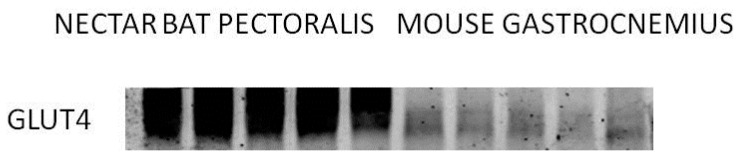
Western blot showing much higher GLUT4 expression in nectar bat (*Glossophaga soricina*) pectoralis muscles (lanes 1–5) than in mouse gastrocnemius (lanes 6–10). 40 µg protein was loaded into each lane. Staining intensity in nectar bat lanes was sixfold greater than in mouse gastrocnemius lanes. Generously provided by Robert Lee-Young and David Wasserman.

A mechanistic requirement for this process to work as hypothesized is a high enough rate of sugar uptake by the flight muscle fibers. In mammalian muscles, rates of glucose transport are increased when the glucose transporter, GLUT4, is translocated from intracellular vesicles to the cell membrane in response to exercise or insulin [44,59]. Nectar bat pectoralis muscles express remarkably high levels of GLUT4 (Figure 5), indicating high capacities for sarcolemmal glucose transport. Along with their high hexokinase *V_max_* values, this makes nectar bats the natural analogues to mice engineered to express high levels of both GLUT4 and hexokinase [44]. In mouse muscles overexpressing GLUT4, transport is enhanced and glucose phosphorylation becomes limiting during exercise. Overexpression of both GLUT4 and hexokinase leads to higher rates of glucose metabolism during exercise than overexpression of GLUT4 or hexokinase alone. The results obtained using mice, through the elegant and powerful combination of genetic, physiological and biochemical approaches [44], were arrived at independently when nectar bats evolved millions of years ago [10].

**Figure 6:**
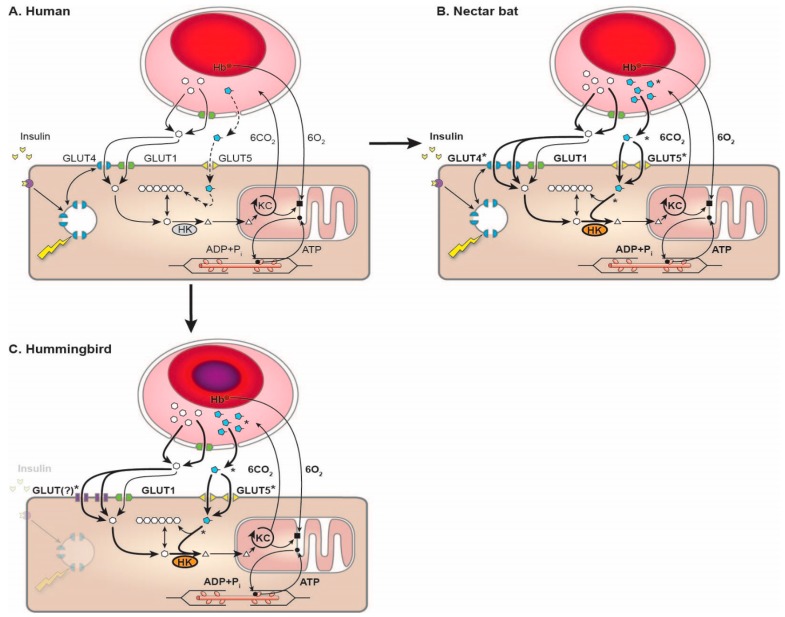
Comparison of mechanisms of muscle uptake and oxidation of circulating glucose and fructose in humans, nectar bats and hummingbirds. (**A**) Pathway for uptake and oxidation of mainly glucose into human skeletal muscle fibers, highlighting key regulatory steps (e.g., sarcollemal transport, regulation of transport capacity via insulin and contractile activity; (**B**) Glucose and fructose uptake and oxidation in nectar bats highlighting enhancements (in bold; those hypothesized are noted by asterisks) to various pathway elements; (**C**) Glucose and fructose uptake and oxidation in hummingbirds highlighting key upregulated steps (bold and asterisks used as in (**B**)). Details regarding functional enhancements are discussed in the main text and in Welch and Chen [63]. Figure from [63].

In a separate experiment of nature, birds evolved to not express GLUT4 and may not even possess the gene for it [60,61]. Consistent with these findings, the flight muscles of ruby-throated hummingbirds (*Archilochus colubris*) show no sign of GLUT4 expression, but do express GLUT1 and GLUT3 [62] (Figure 6).

Experiments involving feeding of hummingbirds with ^13^C-labeled glucose or fructose and determination of δ^13^C of expired CO_2_ reveal hovering flight can be fueled equally well by either sugar [64]. This contrasts with the skeletal muscles of rats and humans that transport and metabolize fructose at much lower rates than glucose [65,66] and, as noted previously, their more limited capacity to directly fuel exercise metabolism with dietary glucose and fructose [67].

Blood glucose concentrations in hummingbirds increase up to 40 mM during repeated bouts of hover-feeding on sucrose solutions and are maintained at 14 mM in the fasted state [63,68]. In contrast, blood glucose concentrations in fasted, resting nectar bats are maintained at about 3 mM. Values increase up to 30 mM after feeding on sucrose solutions and return close to fasted, resting values as a result of exercise after meals [69]. Because both hummingbirds and nectar bats make primary use of the paracellular route in transporting glucose and fructose across the intestinal wall [16,18], differences in flight muscle GLUT expression (Figure 6) offer a likely explanation for the differences in blood glucose kinetics. It appears likely that the ability to restore low concentrations of blood glucose in nectar bats is at least partly the consequence of GLUT4 recruitment and elevated rates of glucose uptake and phosphorylation in the flight muscles in response to insulin and exercise [44]. Birds are generally regarded as “hyperglycemic” relative to mammals and this appears to be due to the absence of GLUT4 and, therefore, the absence of insulin and exercise-stimulated GLUT4 responses in their muscles [61,63]. The ability to fuel muscle metabolism equally well with glucose or fructose [64] underscores the need for further study of sugar metabolism in nectarivorous vertebrates. The roles and mechanisms of regulation of sarcolemmal sugar transporters in hummingbirds during rest and flight, fasting and feeding await elucidation.

## 5. A New Concept: The “Sugar Oxidation Cascade”

**Figure 7:**
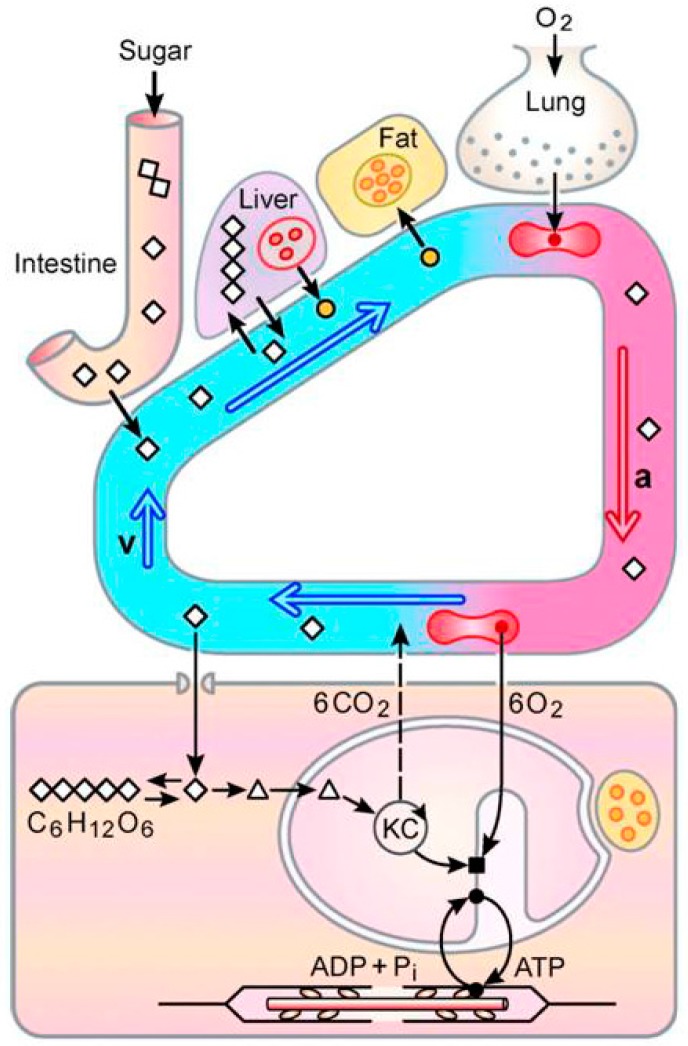
The sugar oxidation cascade provides most of the energy required for flight in hover-feeding hummingbirds and nectar bats. This diagram shows how the sugar oxidation and O_2_ transport cascades operate in parallel in hover-feeding hummingbirds and nectar bats. During hovering flight, >90% of whole-body O_2_ consumption rates are due to flight muscle mitochondrial respiration. In the O_2_ transport cascade, O_2_ travels from the external environment through the respiratory and cardiovascular systems and into muscle mitochondria through a series of convective and diffusive processes at rates determined by muscle energy demands. In the fasted state, mitochondrial respiration is fueled by fatty acid oxidation. During repeated hover-feeding, dietary sugars (twin diamond denotes sucrose; single diamonds denote glucose and fructose) are ingested. Sucrose is hydrolyzed; glucose and fructose cross the intestinal epithelium primarily through a paracellular pathway and enter the blood. Most of the ingested sugar is transported into the flight muscles and broken down. The sugar and O_2_ transport cascades converge in the mitochondria where carbon compounds derived from dietary sugar (pyramids) are oxidized to provide reducing equivalents for respiration and oxidative phosphorylation. Ingested sugars in excess of energetic needs are converted to glycogen (strings of diamonds) and fat (yellow-filled circles). From [3].

We have named the process by which hummingbirds and nectar bats ingest sucrose, glucose and fructose from floral nectars, assimilate glucose and fructose through their intestinal walls, transport and oxidize them in exercising muscles the “sugar oxidation cascade” [3] (Figure 7). It operates in parallel with the oxygen transport cascade [70,71], the process by which animals take in O_2_ from the environment and, via a series of convective and diffusive processes, transports it to exercising muscles where it serves as the terminal electron acceptor. The sugar oxidation and O_2_ transport cascades converge as the carbon derived from recently-ingested sugars is oxidized in flight muscle mitochondria. Hummingbirds and nectar bats are unique among vertebrates in being able to fuel their locomotory muscles during exercise directly with recently-ingested sugar to the extent that their oxidation accounts for most of the V${}_{O_{2}}$ during hover-feeding. In contrast, ingested sugar can directly fuel only about 30%, at most, of the V${}_{O_{2}}$ during exercise in humans [57]. The operation of the sugar oxidation cascade is analogous to aerial refueling in aircraft, wherein fuel “ingested” from a flying tanker is directly combusted to fuel flight.

In both hummingbirds and nectar bats, *V_max_* values for glycogen phosphorylase (Table 1) are sufficient to account for the rates of carbohydrate oxidation required to fuel hovering flight [32,49]. However, metabolic rates during hovering are so high that, if on-board glycogen stores were to serve as the sole fuel for oxidative metabolism in the flight muscles, they would be totally depleted after only several minutes. Of course, this would be unlikely to occur. Instead, we suggest that glycogenolysis during repeated bouts of hover-feeding might function in the flight muscles as it does in mammalian hearts, i.e., glycogen “turns over”, the relative rates of synthesis and breakdown change dynamically, and the process serves to buffer hexose phosphate concentrations [72,73]. Flight muscle power outputs vary as hummingbirds and nectar bats engage in different kinds of flight, e.g., level flight, hovering, aerobatic maneuvers or in response to changes in wing loading and altitude. It seems likely that glycogen resynthesis would occur at rest, between feeding bouts, and that the contribution of glycogenolysis to carbon flux through glycolysis becomes greater under certain circumstances, but only transiently, as in normoxic hearts operating within the range of their physiological power outputs [73]. At this time, the obvious difficulty of assessing rates of muscle glycogenolysis and resynthesis in hummingbirds and nectar bats precludes further discussion beyond the formulation of testable hypotheses.

What might be the advantages derived from direct oxidation of dietary sugar during hover-feeding? One benefit appears to be the direct consequence of the difference between carbohydrate and fatty acid oxidation in ATP yield. Expressed as the P/O ratio, i.e., the number of ATP molecules made per O atom consumed, the oxidation of glucose or glycogen yields a 15% higher P/O ratio than the oxidation of fatty acid [49,50]. This might be advantageous during foraging at high altitude, when hummingbirds must increase muscle power output while experiencing hypobaric hypoxia [49,74]. Another possible advantage is a consequence of the energetic cost incurred when dietary sugar is converted to fat. If this investment were to occur, followed by the oxidation of fat to fuel exercise, then the net energy yield would be 16% lower compared with the direct oxidation of ingested sugar [52]. Direct oxidation of dietary sugar allows more rapid accumulation of fat synthesized from sugar consumed in excess of daily energetic requirements. The rate of fat synthesis appears to be enhanced in nature by foraging behavior that keeps the sugar oxidation cascade turned on and muscle fatty acid oxidation turned off [52,75,76,77].

## 6. Premigratory Sugar Conversion to Fat in Hummingbirds

Certain species of hummingbirds fly long distances during seasonal migrations. Ruby-throated hummingbirds migrate non-stop across the Gulf of Mexico [78]. Rufous hummingbirds make multiple refueling stops as they migrate as far north as Alaska to breed in the summer and as far south as Mexico to escape the cold of winter [79]. As in all other species of flying migrants, hummingbirds make use of fat as the main oxidative fuel for long-term, steady-state flight. Given their high resting and active metabolic rates, the need to maintain daily energy balance (time averaged energy intake = time averaged energy expenditure) is, by itself, a significant challenge. Thus, making an energetic profit (energy intake > energy expenditure) and accumulating fat in preparation for migration is an even more impressive feat. Premigratory fattening becomes even more energetically challenging when higher energetic costs are imposed by low ambient temperature and high elevation [80,81]. Rufous hummingbirds stop to refuel in subalpine meadows during their late-summer, southward migration, where early morning temperatures can be near-freezing. Flight at high elevation requires higher muscle energy expenditure [21] while low temperature increases the energetic cost of thermoregulation [81]. Despite these challenges, hummingbirds have been known to gain about 10% of body mass per day and store up to 40% of body mass in the form of fat during refueling stops [82]. Laboratory experiments involving simulation of such conditions revealed that rufous hummingbirds allowed to perch and to hover-feed at 5 °C for 4 h are able to maintain or gain body mass when provided sucrose concentrations of at least 30%. At 5 °C, more dilute sucrose concentrations result in mass loss (energy intake < energy expenditure) even when the hummingbirds increase their feeding frequencies as they attempt to maintain energy balance [75,83]. At higher ambient temperatures, net fat accumulation can be achieved over a lower range of dietary sucrose concentrations. These experimental results lead to the hypothesis that the coevolution between hummingbirds and the flowering plants that they visit may have resulted in increased sucrose concentrations in floral nectars at higher elevation [83].

## 7. Metabolism in Nectarivorous Animals: Implications for Human Health

Basic research in comparative physiology and biochemistry is usually not done with human physiology or biomedical applications in mind. Instead, it is most often motivated by the desire to explore functional biodiversity across species or to investigate mechanisms of short-term (physiological) and long-term (evolutionary) adaptation. In addition, there is much interest among comparative physiologists in responses to environmental change and their ecological consequences. Nevertheless, studies such as those cited in this brief review illustrate how comparative approaches can benefit biomedical science by complementing traditional approaches, yielding new insights and inspiring new questions.

From an anthropocentric perspective, the idea that certain species of birds and mammals can fuel their extremely high rates of metabolism at rest and during exercise almost entirely with recently-ingested sugars is certainly cause for amazement. The mechanisms by which hummingbirds and nectar bats routinely hover at mass-specific V${}_{O_{2}}$ values about ten- and fivefold higher, respectively, than those of human athletes exercising at V${}_{O_{2}}$*_max_* have been the subject of continuing investigation [2,63,84]. While the paracellular pathway plays a minor role in biomedical models, e.g., [85], it plays a dominant role, accounts for most of the intestinal glucose absorption in nectarivorous animals and operates at rates high enough to supply the fuel requirements of muscles during flight [16,18].

There is current debate concerning the possible roles played by dietary sugars in the development of obesity and diabetes [86,87]. However, what might be a toxic diet for humans serves as the main source of calories for nectarivorous animals. What might appear to be a persistent, severe and potentially harmful hyperglycemia is the natural state of blood glucose homeostasis in hummingbirds [68], animals that are extraordinarily long-lived [88,89] despite their high metabolic rates and small body size. In nectar bats, blood glucose concentrations increase to values high enough to be considered pathological in humans, and are restored to low, resting levels by exercise [69]. A large body of literature concerns how exercise contributes to disease prevention in humans [90,91]. Among the possible mechanisms underlying the beneficial effects of exercise is enhanced myokine production, which leads to autocrine, paracrine and endocrine effects [92,93]. This suggests that the persistent, night-time flight of foraging nectar bats [69] may counteract the negative effects of their sugary diets and hyperglycemia via similar mechanisms.

It has been suggested that honey accounted for a significant fraction of dietary energy intake early in human evolution [94]. Honey, with its high content of glucose (23–41%) and fructose (31–44%) [95], is highly prized and consumed in large quantities by forager societies in various parts of the world [94]. Studies have focused on the Hadza of northern Tanzania whose diet consists of 15% honey [96] but are thin, long-lived and do not suffer from chronic diseases common in Western societies [97]. A surprising finding, based on measurements using doubly labeled water, is that the average total daily energy expenditure of the Hadza hunter-gatherers is similar to that of Westerners. However, the Hadza walk about 6–11 km per day and thereby display higher levels of physical activity than Westerners [98]. Thus, rather than being the result of greater daily energy expenditure, the lack of obesity and metabolic disease among the Hadza may be due to their greater daily physical activity. This is supported by studies involving Western subjects whose walking was reduced to 1300–1500 steps per day for 2 weeks. The reduced activity was found to cause impaired glucose clearance, decreased insulin sensitivity, increased abdominal fat, loss of leg muscle mass and reduction in V${}_{O_{2}}$*_max_* [99,100]. The high fructose content of honey in the Hadza diet is of special significance, given what is known concerning the harmful effects of excessive fructose ingestion [101]. Among Westerners, exercise has been shown to prevent the adverse metabolic effects of high fructose ingestion [102,103]. This is at least partly due to increased fructose oxidation and decreased storage resulting from exercise [104].

Taken together, these data lead to the suggestion that, just as in the case of nectar bats, exercise in humans counteracts the potentially harmful effects of ingestion of large quantities of sugar, particularly fructose. These findings call for further mechanistic studies of sugar metabolism in nectar bats as well as parallel studies on the GLUT4-lacking, chronically-hyperglycemic, nectarivorous hummingbirds. They call renewed attention to Nobel laureate August Krogh’s dictum that “For many problems there is an animal on which it can be most conveniently studied” [105].

## Figures and Tables

**Table 1:** Comparison of enzyme *V_max_* values in locomotory muscles. Data are expressed as µmole substrate converted to product per g, wet mass per minute and temperature-corrected to allow comparison across species. *V_max_* values serve as measures of maximum capacities of flux [38,39] and indicate much higher capacities for glycogen, glucose and long chain fatty acid oxidation in nectar bat and hummingbird pectoralis muscles than in shrew and rat leg muscles. Citrate synthase *V_max_* values serve as relative measures of mitochondrial content [41] and show that nectar bat and hummingbird flight muscles have much higher mitochondrial oxidative capacities than shrew and rat leg muscles.

Enzyme	Nectar Bat ^1^ Pectoralis	Hummingbird ^2^ Pectoralis	Shrew ^3^ Quadriceps	Rat ^4^ Soleus
Glycogen phosphorylase	46.0	59.0	n.a.	10.08
Hexokinase	15.9	18.4	1.10	2.20
Citrate synthase	204.7	448.4	37.0	45.1
Carnitine palmitoyl transferase	6.0	7.2	2.7	0.28

^1^ *Glossophaga soricina*, ^2^ *Selasphorus rufus*, ^3^ *Blarina brevicauda*, ^4^ *Rattus norvegicus*. Data from [32] and references cited therein; n.a. = not available.

**Table 2:** Metabolic fluxes in hovering hummingbirds (*Selasphorus rufus*) and nectar bats (*Glossophaga soricina*). Glucose oxidation rates are estimated in animals performing aerial refueling, i.e., hover-feeding when RQ close to 1.0. Palmitate oxidation rates are estimated in animals hovering in the fasted state with RQ close to 0.7. Both glucose and palmitate oxidation rates are easily accommodated by *V_max_* values for hexokinase and carnitine palmitoyl transferase, respectively (Table 1). Data from [3].

	Nectar Bat	Hummingbird
Whole-body V${}_{O_{2}}$ (mL O_2_ g^−1^ h^−1^)	24.5	33.3
Flight muscle V${}_{O_{2}}$ (mL O_2_ g^−1^ h^−1^)	84.7	119.8
Glucose oxidation rate (µmol g^−1^ min^−1^)	9.1	14.8
Palmitate oxidation rate (µmol g^−1^ min^−1^)	2.0	2.8
